# Preterm Birth Prevention in Appalachia: Restructuring Prenatal Care to Address Rural Health Disparities and Establish Perinatal Health Equity

**DOI:** 10.1089/heq.2020.0064

**Published:** 2021-04-21

**Authors:** Anna Hansen, Mairead E. Moloney, Jing Li, Niraj R. Chavan

**Affiliations:** ^1^ Department of Sociology, University of Kentucky College of Medicine, Lexington, Kentucky, USA.; ^2^ Department of Sociology, University of Kentucky College of Arts and Sciences, Lexington, Kentucky, USA.; ^3^ Center for Health Services Research (CHSR), University of Kentucky College of Medicine, Lexington, Kentucky, USA.; ^4^ Division of Maternal Fetal Medicine, Department of Obstetrics and Gynecology, University of Kentucky College of Medicine, Lexington, Kentucky, USA.

**Keywords:** perinatal health, preterm birth, rural health

## Abstract

**Purpose:** This perspective piece reflects off previously published qualitative work to explore (1) themes surrounding equitable prenatal care in Appalachia and (2) strategies to restructure care delivery in a population with disparate rates of preterm birth (PTB).

**Methods:** This study reflects in-depth interviews with 22 Appalachian women who experienced PTB and 14 obstetric providers.

**Results:** Our findings underscore the need for greater cultural humility in prenatal care, heightened awareness of social determinants of health, and strategic planning to establish equity in birth outcomes.

**Conclusion:** Prenatal care must undergo a paradigm shift to include a comprehensive discussion of cultural humility, social disparities, and health equity.

Preterm birth (PTB) is one of the most pressing public health concerns related to maternal-fetal health in the United States.^1^ PTB, defined as birth before 37 weeks gestation, is associated with increased maternal morbidity, and places neonates at higher risk of mortality and lifelong disability.^2^ PTB complicated ∼1 in 10 deliveries and accounted for nearly 1 in 5 infant deaths in the United States in 2017.^1^ Infants who survive may face a multitude of morbidities, including inadequate pulmonary function, feeding difficulties, cerebral palsy, and developmental delays.^1^

Rates of PTB vary substantially by race, ethnicity, socioeconomic status, and rural residency. Disparate outcomes highlight underlying inequities and the array of risk factors contributing to PTB. Reducing rates of PTB among health disparate populations is a critical public health priority.^3^ Appalachian women constitute an underserved health disparate population disproportionately impacted by PTB.^4^ The March of Dimes consistently assigns Appalachian states a “failing grade” in the national PTB Report Card, due to rates of PTB exceeding the national average.^1,4^ Appalachian women of childbearing age embody multiple PTB risk factors, including higher-than-average rates of diabetes, hypertension, obesity, intimate partner violence, smoking, and substance use disorder in pregnancy.^5–7^

Despite known challenges, there is limited research on PTB in Appalachian women. To begin to address this knowledge gap, we conducted a mixed methods study to evaluate PTB risk stratification, prevention, and management approaches in Appalachian Kentucky. This study was approved by the Institutional Review Board at the University of Kentucky. The Consolidated Framework for Implementation Research (CFIR) informed our study design and in-depth interview questions.^8^ We engaged multiple stakeholders, including Appalachian women with prior PTB and prenatal care providers in rural Appalachia to understand knowledge and practice, and to identify potential points of intervention. Results from 22 in-depth qualitative interviews with Appalachian women and 14 interviews with obstetric providers were previously published, and revealed complex challenges for PTB prevention in Appalachia.^9^ This article reflects on our published work and discusses implications for multilevel interventions.

## Interview Themes Regarding Health Equity

### Cultural humility in prenatal care

If we are to address the public health crisis of PTB effectively, providers must use cultural humility, defined as openness to the cultural values and identity of others (Table 1).^10^ Approaches such as recognizing the limitations of patient circumstances, avoiding assumptions of patients' situations, awareness of implicit biases and cultural stereotypes, and patient-centric communication are critical to optimizing the patient–provider interaction and patient outcomes.^11^ Cultural humility is particularly essential in obstetrics since prenatal care involves sensitive topics, including sexual history and health behaviors (e.g., smoking, substance use, sexual practices), and uncomfortable gynecological procedures. Cultural humility prompts providers to self-critique personal biases, address power imbalances, and develop partnerships with community members.^12^

**Table 1.** Definitions of Key Terms

Health equity strategies	Definition
Cultural humility	A process through which health care provides commit to lifelong self-evaluation and self-critique, to redressing the power imbalances in the patient–physician dynamic, and to developing mutually beneficial and nonpaternalistic clinical and advocacy partnerships with communities on behalf of individuals and defined populations.^12^
Implicit bias training	Acknowledgment that although cultural stereotypes may not be consciously endorsed, their existence influences how information about an individual is processed and leads to unintended biases in decision-making.^12^
Academic detailing	The translation of rigorously reviewed evidence-based information into compelling formats readily accessible for dissemination.^23^
Medical stewardship	A role which may be taken by the health care sector involves facilitating a multisector focus on SDoH and acting as catalysts to involve people and institutions in the promotion of health equity.^24^

SDoH, social determinants of health.

In interviews, Appalachian patients and obstetric providers highlighted the need for cultural humility in prenatal care (Table 2). Patients emphasized the need for respectful and clear communication with providers. Providers relayed the importance of acknowledging patients' constraints and removing judgment from the clinical setting to reduce patients' feelings of stigma. These insights demonstrate that prioritizing patient physical and psychological comfort during routine prenatal encounters is key to patient-centered care.

**Table 2.** Interview Themes and Actionable Steps to Promote Equitable Birth Outcomes

Themes	Patient perspective	Provider perspective	Actionable steps for PTB prevention
Cultural humility	“If there's anything abnormal at all, just go in. It doesn't matter if you think you're bugging them, or anything, your baby's more important than the few minutes that they're wasting on you if nothing's wrong, because it could be something wrong. Because, I didn't think anything was anything was wrong. My water broke, and I was like, ‘Well this is weird,’ so I didn't know. I didn't even think to go to the doctor at first, but it was scary, and I should have. If I hadn't, he might've come really, really early, and there could have been problems, because they did give me a steroid shot. So, without that, he may not have been okay. I guess, don't be afraid to just bug them.” It's also important that they honestly listen to the mother…The doctor came in and the first thing out of her mouth was, “Let me get this straight. You refuse this shot, or you put yourself in your baby at risk for preterm labor because you don't want to get a shot.” And she was just super like snarky about it. I was so upset that I was scared, and in that situation I felt like I couldn't say anything back to her because I knew she had to do an exam. So it was kind of like if I say something and I make her mad, she's liable to be rough about it, and you're already in a vulnerable situation… I feel like sometimes women, mothers are treated like they don't know what they're talking about. But it's your body. So nobody knows your body better than you do.”	“I think have a good rapport with their provider and lots of opportunities to see the provider, the same provider, has the best chance of being effective. And that provider needs to have access to tertiary care centers…you have somebody who has preterm labor, and the weather inclement, that it may not be possible for that patient to get to the tertiary care center because of the weather. So encouraging people to come in and be seen and encouraging them that we don't frown on them coming in. We want them to come in, because it's a partnership. If she's concerned about these things, we want to know, we want those phone calls and we want her to feel good about taking care of herself and her pregnancy by coming in and availing herself to the care that's available. A lot of patients I think feel like the nurses are saying, “Oh no, here comes this patient again.” So we try to work with our nurses to try to encourage them to make the patient feel like this is a partnership. And that she has a real important role in her own care. And she must avail herself to the community hospital and our office. If they're afraid of their doctor, they don't like their doctor, if they feel like their doctor is judging them for whatever reason, you're going to have a much harder time getting these people into…you're more likely to see these people when they're in labor and their labor can't be stopped.” “I guess I try to approach the patient where they are. If there's a potential opening for discussion about what the evidence might be, then I try and take that. I just present the information in a nonconfrontational way, not saying, ‘You're wrong,’ or ‘That person's wrong,’ but try and say, “Well, this is what the experience is, and this is what I have seen, and this is my recommendation,” and try and approach it that way. Try not to be nonconfrontational and also try not to be talking down or paternalistically.”	Train providers in respectful and nonconfrontational communication methods Encourage providers to listen and engage with patients' concerns Create opportunities for patients to see the same provider(s) throughout pregnancy. Familiarize patients with the set-up of the provider group's clinical practice
Utilizing a SDoH framework	“I wish just to have been educated on the situation, on the possibility of that happening. You hear about miscarriages, people having a miscarriage or stillbirth, or anything like that. But as far as an incompetent cervix, you just never hear of it unless it happens to you or somebody close to you. I just feel like they should at least offer some kind of education on it or a pamphlet or just screening for it earlier on in the pregnancy versus having to wait for it to happen to you to figure it out at 20 weeks or something.” “My hospital, they don't have anything like that, to be able to care for a preterm baby… I went into labor…it was like midnight. So, my family, and my husband, they just drove behind the ambulance a hundred miles an hour, all the way there, so it was crazy.” “I mean I do smoke cigarettes but you know, he didn't really tell me not to do that. I guess because I'm pregnant and he's a guy? I mean, when you're talking to a pregnant woman, it's just different. Because I mean, I do get a little aggravated, but I've let him know how much I smoke. I don't care. He's not tell me to quit smoking. I have let him know how much I do smoke, so.”	“I mean part of the problem out here is that there just isn't a lot of access to anything. So there's not a lot of access to medical facilities. There's not a lot of access to meet you even, you know what I mean? There are a lot of people who literally don't have cable or anything like that. Don't drive, don't get out on the roads, don't pass billboards. And so I think that it [preterm birth prevention] really probably needs to start at just smallest scale possible, which I would say would be like places that they go when they get the flu. So primary care offices, we do have a pretty big family medicine network out here, so if they're family docs were screening them for preterm birth before they even got pregnant that would be a big deal. I would think more encouraging them and telling people having a baby at 34 weeks isn't normal that would be a big deal, I think. I would say starting with these primary care offices, these little practices would probably be the best mode of action.” “It's almost like a clan culture. Everybody lives at a holler, and they're all the same family members. You know, you live next to your aunt, and your aunt lives next to your cousin, and your grandpa lives two houses down to your third uncle, or something. It's very clan culture… A lot of patients, they would do anything for you, or their family member. Or they… They're very giving and generous with anything that they have, they just don't have much.” “…We are not a healthy slice of the pie over here in our part of the state. I mean that's why I feel like our preterm rate is kind of what it is… And we're just kind of a unique portion of the state in terms of our hypertensive patients, our obese patients, our diabetic patients, our smokers. We just have less healthy moms in general.” “Our drug addicted mothers are, we are in the heart of the opioid addiction problem in the country, more so than I was when I was in central Kentucky.” “There's not a whole lot of access to specialty care. There's not a lot of education about complications in pregnancy. The general public doesn't know… kind of like avant garde for prenatal care, or drug abusers so… It's such an epidemic that we have patients that get very little prenatal care.”	Collaborate with local primary care providers to initiate early preterm birth education, screening efforts, and health promotion before conception and early in pregnancy Engage, educate, and empower family members to participate in preterm birth prevention efforts Establish referral patterns with regional high-risk pregnancy (perinatology/maternal fetal medicine) subspecialists who can provide high-acuity care Increase availability and accessibility of substance use disorder treatment and recovery services, with an emphasis on the availability of medication-assisted treatment (MAT) for women with opioid use disorder (OUD) in pregnancy.
Establishing equity	“There was nobody—nobody, I don't care who they are—that could afford to drive back and forth and spend enough time with their preemie child that's in the hospital, or whatever the case may be…The only thing that I ever had a problem with [my baby] was where I'm in a treatment facility for Suboxone, and I had been for several years, no problems or anything. No slip-ups. They would not offer or let me stay in the Ronald McDonald house. I guess that that's a rule in a lot of places, where mothers and fathers, for places that they did provide. If they don't want to provide that for someone that's in treatment, then maybe they should make something where parents can stay with their child and be there the whole time, and not have to worry about how they're going to get there and when, everything else.”	“We have what's called a Pregnancy and Beyond program which works with our opiate dependent patients…Anyway, my point is that it's really kind of a team approach. My job is to transcend a lot of it and just be there to support the patients. I'm not the policeman that some of my other partners…I don't play that role where I'm constantly looking for some way to see if there's been some kind of misuse or misdirection of opiate or other medications. I pretty much just support the patient, let the other team, the nurses and other support staff sort of…we talk about the social services and a lot of people are very frightened of the social services, they're afraid they're going to take their baby away. That's a big part of what I do is try to reassure them. Otherwise they drop out, refuse to get prenatal care. That's sort of a long answer, but it's a team approach and I support the patient and try to maintain a therapeutic rapport in which the patient really feels like I'm on their side.”	Integrate social and obstetric services to promote comprehensive prenatal care Enact accessible and affordable support structures (e.g., residential facilities, low-cost housing) for families requiring long-term hospitalization

PTB, preterm birth.

### Utilizing a social determinants of health framework in Appalachia

Greater awareness of social determinants of health (SDoH), the range of social and environmental factors contributing to health, can help providers address disparate health outcomes.^11^ The American Medical Association^13^ and the American College of Obstetrics and Gynecology^11^ have identified SDoH as a vital area of education for physicians. Previous efforts to educate health providers on SDoH have focused largely on socioeconomic and racial health disparities.^14^ Health care providers in Appalachia are faced with a complex web of social forces when caring for patients, including persistent poverty, outmigration, and social isolation.^15^ There are distinctive aspects of Appalachian culture, including isolation and mistrust of outsiders, that present challenges and complexities in seeking care, navigating the health system, and remaining engaged in care over time. There are also cultural assets, including strong traditions of kinship care, which may be positively reinforced within the patient–provider interaction.

Table 2 captures patient and provider insights on challenges and strengths attributable to Appalachian regional culture, and how these characteristics may inform PTB prevention strategies. Participants noted longstanding health disparities preceding pregnancy, the utility of primary care practices, and resilient family structures. Increasing care continuity with a single provider and engaging patients' primary care providers and families may enable obstetric providers to overcome these challenges while embracing the rich and nuanced perspectives Appalachian heritage affords. In addition, clinical efforts to establish perinatal referral networks and increase the accessibility of medication-assisted therapy for opioid use disorder may bolster PTB prevention.

### Establishing equity

Efforts to study PTB in Appalachia must also incorporate strategic efforts to alleviate disparities and promote health equity. Health inequities are differences in health that are systematic, unfair, and avoidable.^16^ Equity in health care requires resource allocation be determined by health needs to alleviate such systematic and avoidable differences.^17^

Health equity in the context of PTB has specific implications for maternal care. Prenatal care in health disparate populations must be prepared to meet both medical and social needs.^18^ Prenatal care models such as group prenatal classes and pregnancy medical homes integrate obstetric and social services, and may advance obstetric health.^18^ Prospective studies have demonstrated reduced PTB and maternal psychosocial stress when patients of health disparate communities receive prenatal care with incorporated social services.^19,20^ Support for integrated social and obstetric services was echoed by providers (Table 2). One patient specifically noted challenges in accessing support structures when simultaneously maintaining recovery from substance use disorders.

## Strategies for Restructuring Prenatal Care in Appalachia

### Implicit bias training

Implicit biases, also known as unconscious biases, refer to how common societal experiences create a shared awareness of stereotypes, which subconsciously influence individuals' perceptions and actions.^14^ All members of society have implicit biases, including health care providers.^14,21^ Past studies have demonstrated implicit biases impact physicians' perceptions and treatment of black individuals, Hispanic individuals, women, elderly individuals, and obese individuals.^14^ Such biases have striking consequences for medical decision-making; physician implicit bias is associated with inequities surrounding proper diagnosis, treatment, and communication.^14^ Minimal research has focused on implicit bias toward rural or Appalachian patients.

Implicit bias curricula may reveal individual's engrained prejudice, and can make individuals aware of unintended involvement in the perpetuation of discrimination.^22^ The importance of implicit bias training is gaining momentum among obstetric health care providers.^23^ Future efforts addressing prejudices toward diverse health disparate patient populations, including rural women and Appalachian women, are needed among prenatal care providers. In interviews, most providers did not speak of patients in stereotypical terms. However, some providers' use of stereotypical language suggests underlying biases (e.g., referring to women with substance use disorder as “drug addicts” or uneducated patients as “unlearned”). Enacting implicit bias training may facilitate actionable steps for PTB prevention outlined in Table 2, namely training providers in respectful communication methods and encouraging providers to engage with patients' concerns. Implicit bias training should be incorporated alongside institutional changes to address bias on multiple levels.

### Academic detailing

Academic detailing (AD) refers to the translation of rigorously reviewed information into compelling formats readily accessible for dissemination.^24^ AD is associated with enhanced adoption of evidence-based practices among providers, optimized patient–provider communication, and cost-effectiveness.^24^ AD offers a mechanism for educating Appalachian prenatal care providers on SDoH and provides language and educational materials to enhance communication with patients.

#### Stewardship and the integration of social services

Equity requires providers to act as responsible stewards for their patient population.^25^ Stewardship describe the roles which may be taken by health care providers in collaboration with other sectors to promote equitable health care.^25^ Revising medical education and training to emphasize SDoH and funding intervention research to address disparities are central priorities for stewardship and the alleviation of maternal and neonatal health disparities.^25^

Traditional clinical programming has been largely unidimensional in its focus on medical need, and prenatal care providers are often unequipped to care for social complexity.^18^ One provider voiced the hopelessness associated with unmet needs of socially vulnerable patients. When discussing issues of confusion and noncompliance among patients at-risk of PTB, they voiced, “The ones that don't [comply], the drug addicts and stuff that just show up once in a while and disappear, you can't do anything about that. No matter how much you try.” Integrating social services alongside prenatal care provides a mechanism for reaching patients whose needs are currently unmet.

## Conclusion

In conclusion, prenatal care delivery in Appalachia has needs that will remain unfulfilled unless the focus of clinical care undergoes a paradigm shift. Findings underscore the need for greater cultural humility in prenatal care, heightened awareness of SDoH and health disparities in clinical practice, and strategic planning to establish equity in birth outcomes. Aligned with these principles, research participants have noted actionable steps toward equitable birth outcomes. Steps include enhancing communication methods, building relationships with patients' family and local primary care clinics, enacting accessible support structures for patients requiring long-term care, and integrating social and obstetric services. Strategies, including implicit bias training, AD, and medical stewardship, may be leveraged to achieve these aims.

## Abbreviations Used

AD: academic detailing
CFIR: Consolidated Framework for Implementation Research
PTB: preterm birth
SDoH: social determinants of health
